# An integrative transcriptomics approach identifies miR-503 as a candidate master regulator of the estrogen response in MCF-7 breast cancer cells

**DOI:** 10.1261/rna.056895.116

**Published:** 2016-10

**Authors:** Jeanette Baran-Gale, Jeremy E. Purvis, Praveen Sethupathy

**Affiliations:** 1 Curriculum in Bioinformatics and Computational Biology, University of North Carolina at Chapel Hill, Chapel Hill, North Carolina 27599, USA; 2 Department of Genetics, University of North Carolina at Chapel Hill, Chapel Hill, North Carolina 27599, USA; 3 Lineberger Comprehensive Cancer Center, School of Medicine, University of North Carolina at Chapel Hill, Chapel Hill, North Carolina 27599, USA

**Keywords:** estrogen receptor α (ERα), gene expression dynamics, microRNAs, miR-503, breast cancer, ZNF217

## Abstract

Estrogen receptor α (ERα) is an important biomarker of breast cancer severity and a common therapeutic target. In response to estrogen, ERα stimulates a dynamic transcriptional program including both coding and noncoding RNAs. We generate a fine-scale map of expression dynamics by performing a temporal profiling of both messenger RNAs (mRNAs) and microRNAs (miRNAs) in MCF-7 cells (an ER+ model cell line for breast cancer) in response to estrogen stimulation. We identified three primary expression trends—transient, induced, and repressed—that were each enriched for genes with distinct cellular functions. Integrative analysis of mRNA and miRNA temporal expression profiles identified miR-503 as the strongest candidate master regulator of the estrogen response, in part through suppression of *ZNF217*—an oncogene that is frequently amplified in cancer. We confirmed experimentally that miR-503 directly targets *ZNF217* and that overexpression of miR-503 suppresses MCF-7 cell proliferation. Moreover, the levels of *ZNF217* and miR-503 are associated with opposite outcomes in breast cancer patient cohorts, with high expression of *ZNF217* associated with poor survival and high expression of miR-503 associated with improved survival. Overall, these data indicate that miR-503 acts as a potent estrogen-induced candidate tumor suppressor miRNA that opposes cellular proliferation and has promise as a novel therapeutic for breast cancer. More generally, our work provides a systems-level framework for identifying functional interactions that shape the temporal dynamics of gene expression.

## INTRODUCTION

Breast cancer remains a prevalent cause of cancer-related death in women worldwide and is categorized into at least five molecular subtypes that differ from each other in terms of biomarkers, etiology, and treatment modalities (Sabatier et al. 2014). By far the most predominant forms of breast cancer are those that stain positive for the estrogen receptor (ER+). The ER, in particular ERα (encoded by the *ESR1* gene), has been widely studied in breast cancer (Creighton et al. 2006; Oh et al. 2006; Zhou and Slingerland 2014). ERα binds to estrogen (usually estradiol or E2), dimerizes, and translocates to the nucleus where it recruits coactivators or corepressors to estrogen response elements (EREs) (Zhou and Slingerland 2014). ERα is thought to be the primary receptor involved in the estrogen response of both normal and breast cancer cells (Higa and Fell 2013).

In response to estrogen, ERα stimulates a transcriptional program involving both coding (Frasor et al. 2003; Rae et al. 2005; Creighton et al. 2006; Gaube et al. 2007; Bourdeau et al. 2008; Chang et al. 2008) and noncoding (Bhat-Nakshatri et al. 2009; Castellano et al. 2009) RNAs. While numerous studies have investigated the coding transcriptional program, only a few studies have investigated the role played by microRNAs (miRNAs). Several miRNAs have been reported to play a role in breast cancer including miR-221/222, which is involved in drug resistance (Rao et al. 2011), and miR-21, which directly targets and regulates numerous tumor suppressors (Zhu et al. 2007, 2008; Frankel et al. 2008; Qi et al. 2009). In one study, miR-375 was identified as an epigenetically deregulated miRNA that amplifies estrogen signaling in ER+ breast cancers (Simonini et al. 2010). Importantly, in that study, the inclusion of miR-375 in a newer microarray probe set allowed the authors to identify a role for miR-375 in ER+ tumors. This highlights the importance of investigating miRNA expression via high-throughput sequencing, which is more sensitive and less biased than microarrays, and could potentially expand the set of miRNAs with potential relevance to the estrogen response.

Although high-throughput sequencing has increased in popularity and decreased in cost over the last decade, it has not been extensively applied toward the study of the gene expression response to estrogen. While microarray studies have identified a working set of estrogen-responsive genes and miRNAs, these studies are limited by several factors. Firstly, microarrays can only identify targets for which probes exist and are subject to cross-hybridization errors (Casneuf et al. 2007). Secondly, to date, most studies have assessed only one or two time points in response to estrogen, which fails to capture the full dynamic responses of ERα targets. ERα is known to cyclically bind to EREs and initiate bursts of transcriptional activity (Shang et al. 2000; Reid et al. 2003), and individual estrogen-responsive mRNAs have been shown to exhibit diverse dynamical patterns of expression following estrogen stimulation (Bourdeau et al. 2008). Finally, to date no study has quantified both coding and noncoding RNAs in the same total RNA in response to estrogen. The estrogen response is highly dependent on the conditions of the study (Wiese et al. 1992), and small changes in experimental design make it difficult to combine multiple studies together. In summary, the body of work on the estrogen response has demonstrated that ERα signaling enacts a dynamic and multilayered gene expression program, but we have very little understanding of how estrogen-stimulated regulatory networks change over time. The study of regulatory networks is greatly enhanced by the inclusion of temporal data, as it expands static interaction diagrams into dynamic models that can uncover complex behaviors, such as the generation of expression thresholds (Mukherji et al. 2011), or the existence of stable points that allow the cell to maintain expression in the absence of continued stimulation.

In this study, we investigate the global response to estrogen stimulation by analyzing paired messenger (mRNA) and miRNA measurements over time in MCF-7 breast cancer cells. We identify three major patterns of gene expression following estrogen stimulation and uncover miR-503 as an important estrogen-induced master regulator of the overall estrogen response. Based on these computational predictions, we confirm experimentally that miR-503 suppresses proliferation in MCF-7 cells, and we identify a new target of miR-503, the oncogene *ZNF217*. These results provide a quantitative understanding of the temporal response of mRNAs and miRNAs to estrogen stimulation, and suggest that miR-503 is a candidate therapeutic target for treatment of breast cancer.

## RESULTS

**FIGURE 1.**
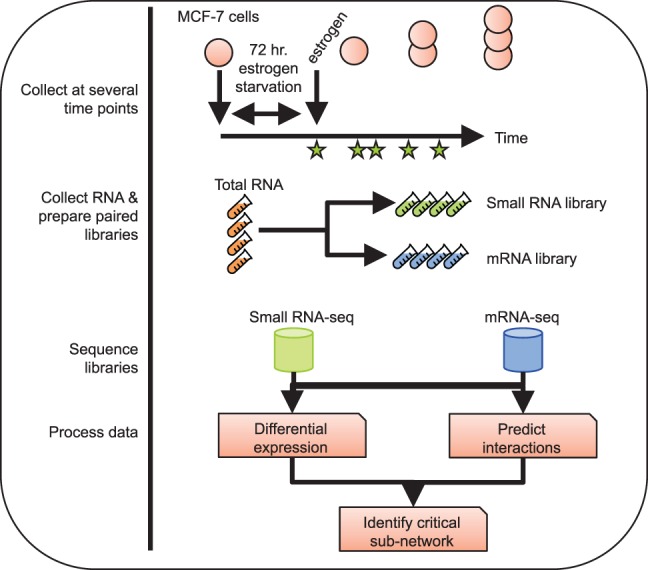
Experimental design. MCF-7 cells were cultured in stripped media for 72 h, then 10 nM E2 was added to the media. RNA was harvested at 0, 1, 2, 3, 4, 5, 6, 8, 12, and 24 h post E2, and paired small RNA and mRNA-seq libraries were generated. Each data set was subject to differential expression analysis, and interactions were predicted between miRNAs and target mRNAs.

To study the dynamics of gene expression in response to estrogen stimulation, we performed a parallel set of time-series measurements for mRNAs and microRNAs (Fig. 1). We cultured MCF-7 cells (a luminal A-type/ER+ cancer cell line) in stripped (estrogen-starved) media for 72 h to synchronize cells in an estrogen-free state. At time zero, we supplemented the media with 10 nM β estradiol (E2) and maintained the cells in this media for 1–24 h. At each of 10 time points (hourly from 0 to 6 h after E2, and 8, 12, and 24 h after E2), with three independent biological replicates for each, cells were harvested and used to prepare both small RNA and poly(A)^+^ RNA libraries from the same total RNA sample for high-throughput sequencing. The poly(A)^+^ RNA libraries had an average read depth of ∼65 million reads (>90% of reads aligned uniquely), and the small RNA libraries had an average read depth of ∼32 million reads (∼90% of reads aligned) (Supplemental Table S1). The expression levels of selected genes and miRNAs were confirmed by RT-qPCR (Supplemental Fig. S1).

### Dynamics of estrogen-regulated mRNAs

**FIGURE 2.**
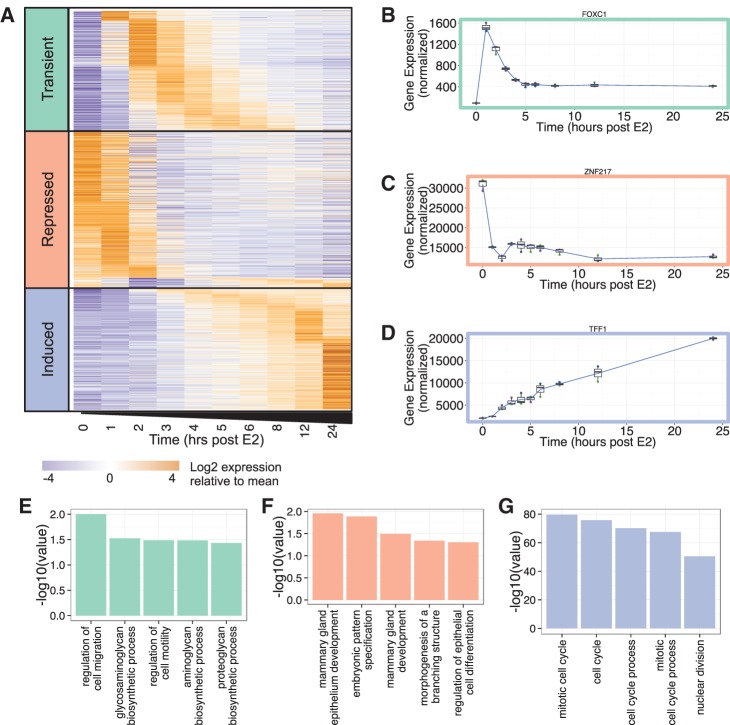
Gene expression response to estrogen stimulation. (*A*) The expression profile of 1546 mRNAs that have a significant (adjusted *P*-value <0.05) greater than or equal to twofold change at one or more time points in response to estrogen stimulation, and a mean normalized expression of at least 500 across the time series. Genes are clustered into three classes (transient, repressed, and induced). (*B*) Expression profile of *FOXC1*, an example of a transient gene. (*C*) Expression profile of *ZNF217*, an example of a repressed gene. (*D*) Expression profile of *TFF1*, an example of an induced gene. All plots show the mean of three biological replicates as a blue line with a box and whisker plot showing the variation in normalized expression among the replicates. (*E*) Selected results from Gene Ontology analysis of genes in the transient class. (*F*) Selected results from Gene Ontology analysis of genes in the repressed class. (*G*) Selected results from Gene Ontology analysis of genes in the induced class.

Differential gene expression was calculated for each time point relative to time zero. Those genes with a mean normalized expression count across the time series of at least 500, and a significant (adjusted *P*-value <0.05) greater than or equal to twofold change relative to time zero at any time during the 24 h, were considered to be estrogen-responsive. In total, 1546 genes met these criteria. Using this condensed data set, we then examined the dynamics of gene expression. By clustering the time series, we were able to stratify the 1546 genes into three temporal patterns of gene expression (Fig. 2A; Supplemental Table S2). The first class includes 468 genes that show transient induction in response to estrogen. For example, the gene encoding the forkhead transcription factor, *FOXC1*, which is an important biomarker of basal-like breast cancer (Jensen et al. 2015), is lowly expressed at time zero, exhibits a brief but significant increase in expression, and then stabilizes at a new equilibrium state ∼5 h after estrogen stimulation (Fig. 2B). Both peak times and peak widths are variable within this class. Approximately 11% of the genes in this list have previously been identified (by meta-analysis of microarray studies) to be up-regulated at ∼4 h after estrogen stimulation (Jagannathan and Robinson-Rechavi 2011). The second class includes 608 genes that exhibit overall decreases in expression over time. These “repressed” genes include *ESR1* (gene encoding ERα), *ERBB2*, *GATA3*, and *ZNF217* (Fig. 2C)—all critical genes to the etiology of breast cancer (Sørlie et al. 2001; Usary et al. 2004; Oh et al. 2006; Cohen et al. 2015). *ZNF217*, a notable member of this class of genes, is a Krüppel-like finger (KLF) protein that acts as a transcriptional regulator that amplifies the estrogen response in breast cancer (Cohen et al. 2015) and has been identified as a biomarker of poor survival in patients with Luminal A (ER+) breast tumors (Frietze et al. 2014). Approximately 40% of the genes in the repressed class were previously identified as down-regulated at 24 h after estrogen stimulation (Jagannathan and Robinson-Rechavi 2011). Finally, 470 genes are induced by estrogen stimulation. Included in this class are *TFF1* (Fig. 2D; Supplemental Fig. S1) and *CTSD* (Supplemental Fig. S1), for which there exists a detailed time course of ChIP data showing cyclic occupancy of ERα on their promoters (Shang et al. 2000; Reid et al. 2003). Approximately 56% of these genes have been previously identified as up-regulated at 24 h post-estrogen stimulation, including known breast cancer genes *BRCA1*, *BRCA2*, and *E2F1* (Suter and Marcum 2007)*.* Gene ontology (GO) analysis indicates that each of these three classes of genes is enriched for distinct functional categories (Fig. 2E–G). The transient group of genes shows enrichment for GO terms dealing with cell migration and motility (Fig. 2E), the repressed group for terms involving mammary development and differentiation (Fig. 2F), and the induced category for functions relevant to cell cycle progression (Fig. 2G). Taken together, this analysis reveals a complex and multilayered gene expression program with different temporal patterns of expression associated with distinct cellular functions.

**FIGURE 3.**
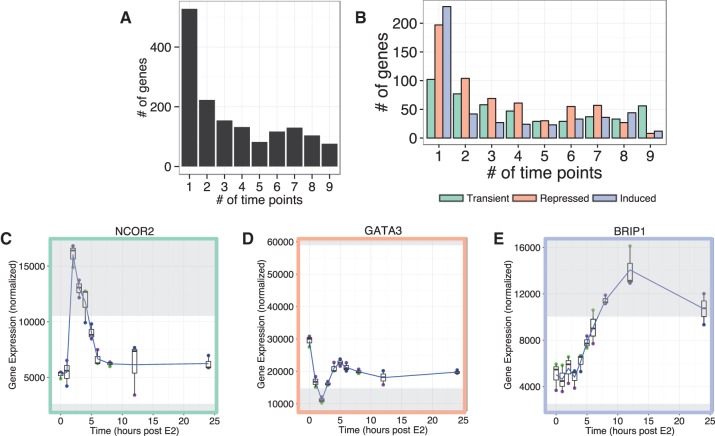
Most genes reach greater than or equal to twofold change at few and disparate time points. (*A*) The number of time points during the 24 h collection for which each of the 1546 estrogen-responsive genes reaches a greater than or equal to twofold change from time zero. (*B*) The number of time points during the 24 h collection for which genes within the three classes reach a greater than or equal to twofold change from time zero. (*C*) The expression profile of *NCOR2*, a representative gene from the transient class, reaches greater than or equal to twofold change from time zero at three of the time points. (*D*) The expression profile of *GATA3*, a representative gene from the repressed class, reaches greater than or equal to twofold change from time zero at one time point. (*E*) The expression profile of *BRIP1*, a representative gene from the induced class, reaches greater than or equal to twofold change from time zero at three of the time points. All plots show the mean of three biological replicates as a blue line with box and whisker plots showing the variation in normalized expression among the replicates, and regions greater than or equal to twofold different than time zero are shaded in gray.

It is important to note that our high-resolution temporal analysis identified many estrogen-responsive genes that would have been missed had we taken a more conservative approach and examined only one or a few time points. We find that 59% (*n* = 905) of the estrogen-responsive genes exhibit greater than or equal to twofold change at three or fewer time points; furthermore, 34% (*n* = 528) of the estrogen-responsive genes exhibit greater than or equal to twofold change at only a single time point (Fig. 3A). This observation that a large proportion of estrogen-responsive genes is significantly altered at only a few time points, and not necessarily at the same time points, is evident in all three classes of response patterns (Fig. 3B). These data highlight the added value of a high-resolution temporal analysis. For example, consider nuclear corepressor 2 (*NCOR2*), which is a member of the same nuclear receptor super-family as ERα, and has been associated with early tumor recurrence in breast cancer (Smith et al. 2012). *NCOR2* is only greater than or equal to twofold up-regulated at three of the 10 time points analyzed (2–4 h post-estrogen stimulation) (Fig. 3C, adjusted *P*-value = 4 × 10^−17^ at 2 h, 1 × 10^−11^ at 3 h, 2 × 10^−9^ at 4 h post E2). A study designed to assess estrogen-responsive genes at 12 or 24 h post-stimulation would detect virtually no difference in gene expression levels of *NCOR2*. Additionally, within the repressed class, a group of genes drops significantly in expression in the first 2 h after estrogen stimulation, but then recovers at an expression level roughly 60% of the expression at time zero (Supplemental Fig. S2A). Both *GATA3* (Fig. 3D) and *ESR1* are members of this class, and these encode transcription factors that not only regulate each other (Eeckhoute et al. 2007) but also coregulate many target genes (Kong et al. 2011). *GATA3* is greater than or equal to twofold down-regulated only at 2 h post-estrogen. Nearly half of the genes in the induced class reach a greater than or equal to twofold up-regulation (adjusted *P*-value <0.05) at only a single time point (Fig. 3B). Finally, *BRIP1* (BRCA1 interacting protein C-terminal helicase 1; Fig. 3E) is an example of a gene in the induced class that is detected as differentially expressed at only three time points (all after 8 h post-estrogen stimulation). These observations demonstrate the dynamic nature of gene expression in response to estrogen and the importance of collecting and analyzing high-resolution time-series data to fully capture these dynamics.

We also identified two groups of genes displaying opposite temporal responses to estrogen. The first group of 170 genes displays a temporal response to estrogen similar to that of *GATA3* (Supplemental Fig. S2A). The importance of both GATA3 and ERα in regulating the estrogen response has been established (Kong et al. 2011); therefore, these 170 genes may include other members of this regulatory network, either additional coregulators of ERα or genes regulated by these transcription factors. The second group includes 118 genes that exhibit a temporal response to estrogen that is opposite of the *GATA3*-like genes (Supplemental Fig. S2B). The opposite temporal responses of these groups suggest a possible inhibitory relationship between members of the two groups. To further explore this inverse relationship, we sought to identify potential negative regulators of *ESR1* or *GATA3* within the anti-*GATA3* group. To that end we assessed the correlation between both *ESR1* and *GATA3* and genes within the anti-*GATA3* group in The Cancer Genome Atlas (TCGA) breast cancer data set (https://tcga-data.nci.nih.gov/tcga/). The transcription factor *FOXC1* (Fig. 2B), an important biomarker of basal-like breast cancer (Jensen et al. 2015), is a member of the anti-*GATA3* group and is anticorrelated with *GATA3* expression in TCGA data (Supplemental Fig. S2C; Pearson's *r* = −0.602). This analysis confirms previous findings that FOXC1 and GATA3 are involved in a switch (Supplemental Fig. S2D) between basal-like and luminal-like expression programs in breast cancer (Tkocz et al. 2011), and indicates that the high-resolution time-series data may identify novel factors that underlie this switch.

### Dynamics of estrogen-regulated miRNAs

To understand how the temporal gene expression patterns are regulated, we next sought to characterize the dynamics of miRNA expression in response to estrogen. Small RNA-seq data were processed as previously described (Baran-Gale et al. 2013) to identify robustly expressed miRNAs and their isoforms (isomiRs). Resulting miRNA counts were normalized using a reads-per-million-mapped (RPMM) transformation. We detected 308 miRNAs with a mean expression of at least 50 RPMM across all samples (Supplemental Table S3). Consistent with previous studies of MCF-7 cells (Bhat-Nakshatri et al. 2009), among the most highly expressed miRNAs in the data set are miR-21-5p, miR-200c-3p, the let-7 family, and miR-93-5p.

**FIGURE 4.**
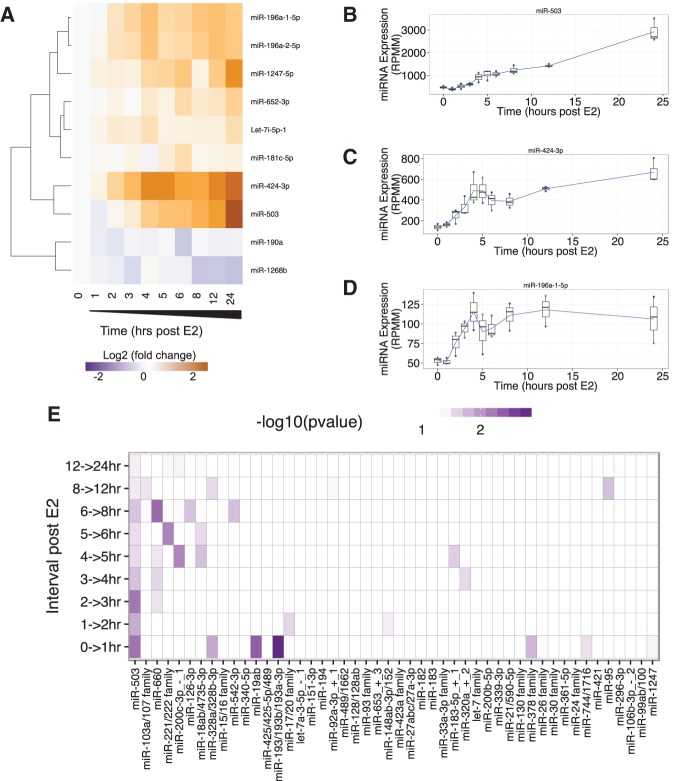
miRNA expression response to estrogen stimulation. (*A*) The expression profile of 10 miRNAs that have a ≥1.5 mean fold change of at least one time point in response to estrogen stimulation and a mean normalized expression of at least 50 RPMM. The expression profiles of miRNAs were clustered using a hierarchical clustering method. (*B*) Expression profile of the strongest responding miRNA, miR-503. (*C*) Expression profile of miR-424-3p. (*D*) Expression profile of miR-196a-1-5p. All plots (*B*–*D*) show the mean of three biological replicates as a blue line with box and whisker plots showing the variation in normalized expression among the replicates. (*E*) This plot shows the −Log_10_(uncorrected *P*-value) of enrichment for each miRNA family among the genes that is characteristic of the change in expression between each time interval. miRNA families on the *x*-axis are sorted by decreasing significance (sum across all time intervals).

To identify estrogen-responsive miRNAs, the expression of each miRNA was normalized to the mean of the three replicates at time zero. Of the 308 expressed miRNAs, 10 exhibited a fold change of at least 1.5 (uncorrected *P*-value ≤0.05) at some time point during the 24 h (Fig. 4A), and five miRNAs had a fold change greater than two (uncorrected *P*-value ≤0.05; miR-503, miR-424-3p, miR-1247-5p, miR-196a-1-5p, and miR-196a-2-5p). The miRNA with the highest fold change following estrogen stimulation is miR-503 (Fig. 4B), with an approximately sixfold increase by 24 h post-estrogen stimulation. Interestingly, the second-most strongly increased miRNA is miR-424-3p (Fig. 4C), which is encoded on the same primary transcript as miR-503. Although literature corresponding to miR-424 usually refers to miR-424-5p, in our data miR-424-3p is more consistently expressed across replicates, has a higher mean expression across the time series, and has a greater fold increase than miR-424-5p. miR-1247-5p exhibits a threefold increase in response to estrogen stimulation, and both paralogs of miR-196a-5p (Fig. 4D) are approximately twofold increased by 4 h after estrogen stimulation.

### Computational prediction of miRNA–mRNA regulatory interactions

We next explored the potential regulatory interactions between miRNAs and mRNAs in the temporal response to estrogen using our previously published miRNA target site enrichment algorithm, miRhub (Baran-Gale et al. 2013). miRhub identifies candidate master miRNA regulators by identifying those miRNAs that are predicted to target and regulate a gene set of interest significantly more than expected by chance. We sought to not only identify potential miRNA–mRNA regulatory interactions throughout the entire time course but to determine the specific time points at which these interactions were most significant. To do this, we used the characteristic directions method (Clark et al. 2014) to identify the sets of genes whose combined expression best distinguish the expression profiles between consecutive time points (Supplemental Table S4). We then assessed all expressed miRNA families to determine whether any are candidate “master regulators” of these sets of “characteristic genes.” Using this approach, miR-503 consistently emerged as the most significant candidate master miRNA regulator (Fig. 4E). It was particularly prominent at time points 1, 2, 3, and 4 h post-treatment. Thus, miR-503 is both the most estrogen-responsive miRNA as well as the miRNA with the largest predicted impact on the dynamic gene expression response to estrogen.

**FIGURE 5.**
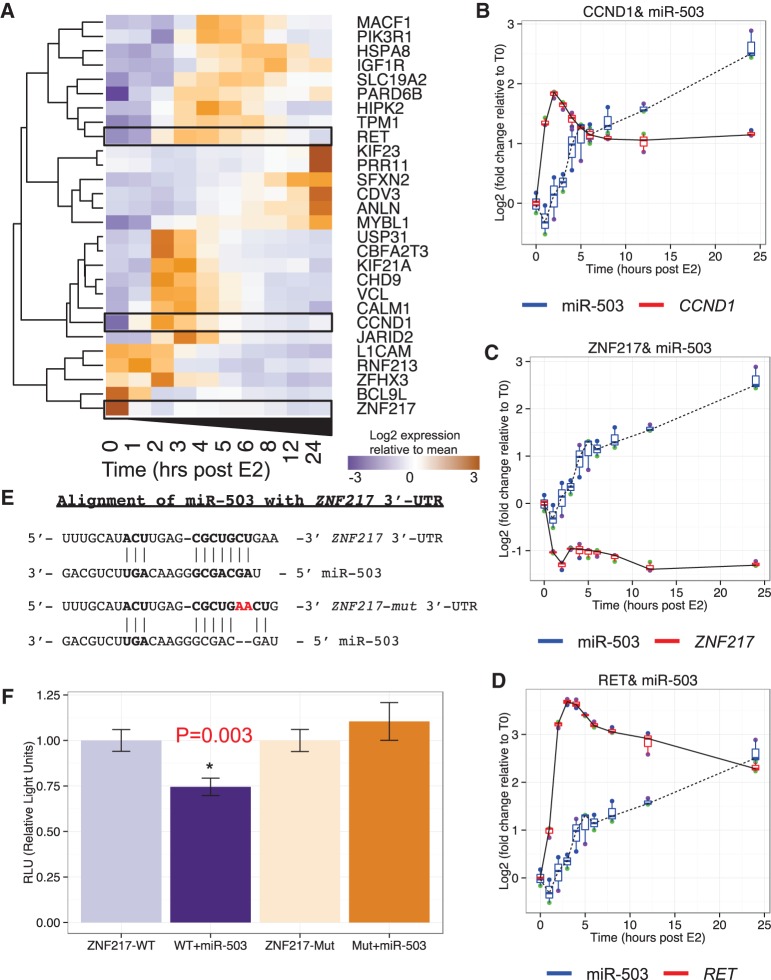
Potential miR-503 targets. (*A*) The expression profile of 28 miR-503 targets mRNAs that are characteristic of the difference in gene expression between consecutive time points. The expression profiles of miRNAs were clustered using a hierarchical clustering method. (*B*) Expression profile of miR-503 and validated target, *CCND1*. (*C*) Expression profile of miR-503 and predicted target, *ZNF217*. (*D*) Expression profile of miR-503 and predicted target, *RET.* All plots (*B*–*D*) show the mean of three biological replicates as a blue line with box and whisker plots showing the variation in log_2_ (fold change) between replicates. (*E*) miR-503 target site in the *ZNF217* 3′-UTR. A dual-luciferase reporter was used to validate the response of *ZNF217* to miR-503. The reporter was mutated by inserting two A's (red) to disrupt the seed region binding of miR-503. (*F*) Response of the *ZNF217* reporter and mutant reporter with and without 10 nM miR-503 mimic. Significance assessed using a Student's *t*-test.

Following our identification of miR-503 as a potential master regulator of the estrogen response, we next sought to identify potential targets of miR-503. Our miRNA target site enrichment analysis revealed 28 genes that are predicted targets of miR-503 (Fig. 5A). One of the predicted targets, *CCND1* (Fig. 5B), has already been validated as a target of miR-503 and the repression of *CCND1* by miR-503 has been reported to inhibit proliferation in breast cancer cell lines (Long et al. 2015). Another predicted target, *ZNF217* (Fig. 5C), has not been reported as a miR-503 target but was recently identified as both a biomarker and an oncogene in breast cancer (Cohen et al. 2015). As a final example, *RET* (Fig. 5D) is a proto-oncogene that is reported to be transcriptionally up-regulated in numerous human cancers (Plaza-Menacho et al. 2014). Other genes within the list of potential miR-503 targets provide potentially interesting insights into the estrogen response regulatory network in breast cancer and warrant further study (Fig. 5A).

Among these predicted miR-503 targets, *ZNF217* is of particular interest because of its known mechanistic role in the estrogen response. ZNF217 binds to many of the same promoters as the three transcription factors that coordinate the overall response to estrogen stimulation (ERα, GATA3, and FOXA1) (Kong et al. 2011; Frietze et al. 2014). Additionally, the C terminus of ZNF217 physically binds to the hinge domain of ERα and enhances recruitment of ERα to EREs (Nguyen et al. 2014). We carried out ChIP-X enrichment analysis (ChEA) (Chen et al. 2013) and found that ZNF217 binding sites are overrepresented in estrogen-responsive genes in our data set (Supplemental Fig. S3). During the first 4 h post-estrogen treatment, the behavior of *ZNF217* bears a strong resemblance to that of *GATA3*. However, unlike *GATA3*, *ZNF217* fails to recover from greater than twofold repression and remains twofold down-regulated for the rest of the time course. In fact, the first time point that *ZNF217*’s fold difference in expression deviates from that of *GATA3* is at 4 h post-estrogen stimulation. At that same time point (hour 4), miR-503 reaches an approximately twofold increase relative to time zero. Taking together these compelling observations with the etiological relevance of ZNF217 to breast cancer, we selected *ZNF217* as a potential target of miR-503 for further investigation.

### The role of miR-503 and *ZNF217* in cellular proliferation and breast cancer

To validate the repression of *ZNF217* by miR-503, we carried out 3′-UTR reporter gene assays. Specifically, we cotransfected miR-503 in MCF-7 cells with dual-luciferase expression vectors containing a Renilla luciferase reporter gene (internal control) and a Firefly luciferase reporter gene linked to either the wild-type *ZNF217* 3′-UTR or a mutated version of the *ZNF217* 3′-UTR reporter with two adenosines inserted between the bases opposite of nucleotides 3 and 4 in the predicted miR-503 target site (Fig. 5E). This mutation abolishes the perfect match to the miR-503 seed region, and therefore is expected to compromise the efficacy of miR-503 targeting. We found that miR-503 significantly (*P* = 0.003) reduces the relative levels of the wild-type *ZNF217* 3′-UTR reporter, whereas the mutation in the miR-503 target site rescues this effect completely (Fig. 5F). Additionally, overexpression of miR-503 in MCF7 cells resulted in significant repression (∼30% loss, *P* = 0.026) of ZNF217 protein at 48 h after transfection (Supplemental Fig. S4). Together, these results indicate that miR-503 represses *ZNF217* in MCF-7 cells via direct targeting of its 3′-UTR. Furthermore, ZNF217 has a demonstrated role in promoting proliferation in several models of breast cancer. Specifically, silencing of *ZNF217* results in reduced proliferation in several breast cancer cell lines (including MCF-7), and overexpression of *ZNF217* increases proliferation in the same cell lines as well as in xenograft tumors (Thollet et al. 2010). The established role of *ZNF217* as an oncogene combined with these findings strongly support miR-503 as a candidate tumor suppressor in breast cancer.

The role of miR-503 in opposing proliferation has been very recently investigated in breast cancer (Long et al. 2015; Polioudakis et al. 2015), prostate cancer (Guo et al. 2015), and osteosarcoma (Chong et al. 2014). One previous study in MCF-7 cells demonstrated that overexpression of miR-503 was able to inhibit cell cycle progression through repression of *CCND1* (Long et al. 2015). Based on our findings that miR-503 targets *ZNF217*, which promotes cell cycle progression (Li et al. 2014), we hypothesized that the anti-proliferative effect of miR-503 resulted from a G1 arrest. To test this prediction, we transiently transfected MCF-7 cells with miR-503 mimic (50 nM). Cells were pulsed for 2 h with EdU and fixed at 48 and 72 h after transfection. For each cell, the total EdU signal was plotted against the total DNA signal, and cell cycle stage was assigned. We found that MCF-7 cells transfected with miR-503 have a significantly reduced percentage of cells in S phase compared to mock transfection (Supplemental Fig. S5A). This observation was observed at both 48 h (*P*-value = 0.01) and 72 h (*P*-value = 0.03) after transfection with miR-503 (Supplemental Fig. S5B). We validated the anti-proliferative effect of miR-503 by showing a reduced amount of Ki67 protein (Supplemental Fig. S6).

Furthermore, data from patient cohorts confirm that low expression of *ZNF217* (Supplemental Fig. S7A; Győrffy et al. 2009; Frietze et al. 2014) and high expression of miR-503 (Supplemental Fig. S7B; Antonov 2011; Lyng et al. 2012; Antonov et al. 2013) in breast cancer is significantly associated with improved survival (*P* = 0.038 for *ZNF217* and *P* = 0.000437 for miR-503). Together, these data confirm that miR-503 inhibits proliferation in MCF-7 cells, and that the oncogene *ZNF217* is a novel target of miR-503. These findings motivate further mechanistic studies to determine the extent to which the anti-proliferative role of miR-503 is mediated through suppression of *ZNF217*.

## DISCUSSION

An extensive body of literature exists detailing the importance of ERα in breast cancer, both as a biomarker of cancer severity and as a therapeutic target. Additional studies have highlighted the importance of various miRNAs in the etiology of breast cancer (Bhat-Nakshatri et al. 2009). In this study, we provide the first detailed high-throughput sequencing time course of paired mRNA and miRNA expression in response to estrogen stimulation in MCF-7 cells. These data have provided a wealth of insight into the dynamics of the estrogen response. We observe similar temporal responses for genes that encode the transcription factors previously reported to be involved in positive feedback loops (ERα and GATA3; Eeckhoute et al. 2007) and opposite temporal responses for genes encoding transcription factors reported to inhibit each other (GATA3 and FOXC1; Tkocz et al. 2011). These temporal relationships allow us to make inferences about the estrogen-stimulated regulatory network and enhance our understanding of the timing of the cascade of signaling stimulated by estrogen in breast cancer.

This study is one of the few to investigate the temporal response of RNAs to stimuli in breast cancer, and is the first sequencing based study to investigate the matched temporal response of coding and noncoding RNAs to estrogen stimulation in breast cancer. A previous study used microarrays to investigate the response of genes and miRNAs to estrogen stimulation (Cicatiello et al. 2010) but, interestingly, did not identify miR-503 as a significantly expressed miRNA. This discrepancy underscores the importance of using high-throughput sequencing and fine-grained time resolution to study the dynamics of gene expression.

**FIGURE 6.**
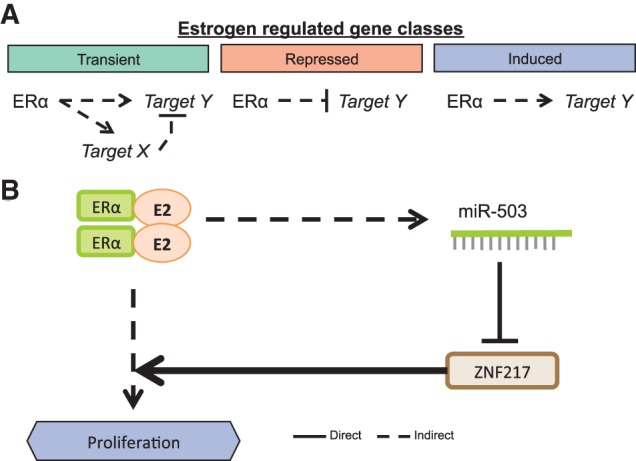
Summary. (*A*) Potential mechanism behind regulation of the three classes of estrogen-responsive genes. (*B*) Summary mechanism showing the interaction of the estrogen-responsive genes *ESR1* and *ZNF217* and the estrogen-responsive miRNA miR-503.

Estrogen stimulation induces a dynamic and varied response in 1546 mRNAs and 10 miRNAs. The transient class of estrogen-stimulated mRNAs contains genes that peak for various lengths of time and at different time points following estrogen stimulation (Fig. 6A). Such behavior may be due to an incoherent feed-forward loop architecture, wherein both a target gene and its repressor are activated leading to a pulse in gene expression (Alon 2007). The repressed category also exhibits significant variation, with a large group of genes behaving similarly to *GATA3* (an initial drop in expression followed by a recovery at a new baseline expression level). Finally, the induced class of estrogen-responsive mRNAs appears to either continually increase throughout the experiment as *TFF1* (Fig. 2D) or level off at some new higher expression level. Among the estrogen-responsive miRNAs, miR-503 emerges as the most strongly responsive miRNA, though others that are greater than twofold changed (miR-424-3p, miR-1247-5p, miR-196a-1-5p, and miR-196a-2-5p) warrant further investigation as well.

Interestingly, we also noted that a group of transient mRNAs has the exact opposite expression pattern to the *GATA3*-like group of repressed mRNAs (an initial increase followed by a reversion to a lower but still up-regulated expression level). A representative example of this group is *FOXC1* (Fig. 2B), an important regulator of Basal-like breast cancer and a repressor of *GATA3* (Tkocz et al. 2011). *FOXC1* and *GATA3* appear to be involved in a double-negative-feedback loop (mutual inhibition) that may influence the transition between luminal-like and basal-like tumor phenotypes (Supplemental Fig. 2D). Tkocz and colleagues showed that knockdown of *GATA3* in luminal cells resulted in the expression of basal-like markers and cell morphology, while knockdown of *FOXC1* in basal-like cells resulted in the expression of luminal markers and cell morphology (Tkocz et al. 2011). It is interesting to observe this pulse in a master regulator of the basal-like phenotype in response to estrogen. One explanation for this behavior is that the transition from estrogen-free media to estrogen-rich media may displace the ligand unbound estrogen receptor from its target sites and allow expression of genes that were repressed by the receptor in the absence of ligand. The introduction of estrogen would presumably then lead to the ligand-bound estrogen receptor repressing those same genes.

Among the targets (direct or indirect) of the ERα regulatory circuit are *ZNF217* and miR-503 (Fig. 6B). ZNF217 is a transcriptional regulator that works together with ERα to amplify the estrogen response in breast cancer (Cohen et al. 2015). However, despite the association of ERα with the promotion of proliferation, *ZNF217* is repressed in response to estrogen stimulation in our data, as well as in previously published data (Cicatiello et al. 2010; Jagannathan and Robinson-Rechavi 2011). Consistent with the repression of *ZNF217*, the genes encoding the main transcription factors involved in the estrogen response (*ESR1* and *GATA3*) are also repressed at the early time points. Together, these data indicate that there may be a built-in mechanism to (i) avoid the ZNF217-induced attenuation of the estrogen response and (ii) limit further stimulation of the estrogen response pathway. In this study, we identify three major patterns of gene expression following estrogen stimulation: (i) transient, (ii) induced, and (iii) repressed. Among the genes repressed by estrogen stimulation is the oncogene *ZNF217*, which has been shown to enhance proliferation in ovarian (Li et al. 2014) and breast cancer (Thollet et al. 2010). Additionally, we have shown that the estrogen-induced miRNA, miR-503, targets the 3′-UTR of *ZNF217* and that while miR-503 is induced in response to estrogen, the oncogene *ZNF217* is reduced. In support of this relationship, data from breast cancer patient cohorts show that high expression of *ZNF217* is associated with worse survival (Győrffy et al. 2009; Frietze et al. 2014), while high expression of miR-503 is associated with improved survival (Antonov 2011; Lyng et al. 2012; Antonov et al. 2013). Furthermore, while this manuscript was under review, another study was published demonstrating that the GATA3-driven expression of miR-503 represses *ZNF217* in prostate cancer (Jiang et al. 2016). Together, these observations point to the complexity of the estrogen-signaling network and further highlight the beneficial aspects of the estrogen response. Several studies have recently shown that miR-503 may be a potent tumor suppressive miRNA. One such study showed that miR-503 targets *CCND1* and reduces proliferation in both MCF-7 and MDA-MB-231 cells (an ER [−] Claudin-low model) (Long et al. 2015). Others have shown that miR-503 reduces proliferation and metastasis in prostate cancer (Guo et al. 2015), in osteosarcoma (Chong et al. 2014), and in hepatocellular carcinoma (Li et al. 2015). Furthermore, loss of miR-503 has been reported in several human cancers and is associated with poor prognosis in cervical cancer (Yin et al. 2015). The induction of miR-503 in response to estrogen stimulation has anti-proliferative effects, likely through its repression of both *CCND1* and *ZNF217*. Combined with the information that miR-503 is down-regulated in human cancers, these results indicate that miR-503 presents a new candidate therapeutic option for the treatment of breast cancer. Moreover, this study highlights the benefits of integrative analysis of the global response of model systems to relevant stimuli. Such studies can direct further research in primary tumors and disease models.

## MATERIALS AND METHODS

### Cell culture and estrogen treatment

The Perou Laboratory at UNC Chapel Hill generously provided the MCF-7 cells used in these experiments. Cells were cultured in Dulbecco's modified Eagle's medium (DMEM) with Ham's F-12 nutrient mixture, 15 mM HEPES, and sodium bicarbonate (Sigma; #D6434). Media was supplemented with 10% charcoal-stripped serum (Sigma; #F6765) and 5% GlutaMax (Gibco; #35050-061). For each biological replicate, a single plate of cells was split into 10 separate cell culture plates (one for each time point). Cells were maintained in the stripped serum media for 72 h, then the time zero batch of cells was scraped from the plate, pelleted and flash frozen in ethanol dry ice slurry. For the remaining cells (other time points), media supplemented with 10 nM β-estradiol (Sigma; #E2758) was added at time zero, and cells were collected at 1, 2, 3, 4, 5, 6, 12, and 24 h after addition of E2-media.

### Sequencing and differential expression analysis

MCF-7 cells were lysed and RNA was isolated using the Norgen Total RNA Purification Kit (#17200). Only samples with an RNA Integrity Number (RIN) of 9 or higher, as measured by Agilent Bioanalyzer 2100, were considered for further analysis. Small RNA libraries were generated using the Bioo Scientific NEXTflex V2 kit (#5132-03) and sequenced on the Illumina HiSeq 2000 platforms. Small RNA-seq reads were trimmed using cutAdapt (−O 10 –e 0.1) (Martin 2011) to remove remnants of the 3′-adapter sequence, then the first four and last four nucleotides of small RNA-seq reads were trimmed to remove the degenerate nucleotides in the adapters. Subsequent mapping of trimmed reads to the human genome and miRNA/isomiR quantification were performed exactly as previously described (Baran-Gale et al. 2013). The threshold used to classify miRNAs as robustly expressed was set at a mean of 50 RPMM across the time series.

RNA-seq libraries were generated from the same total RNA isolated above using the Illumina TruSeq stranded mRNA library prep kit (#RS-122-2101) and were sequenced on the HiSeq2500 (2 × 50). Reads were aligned to the human genome (hg19) using MapSplice (2.1.4) (Wang et al. 2010), and transcript abundance was quantified using RSEM (v1.2.9) (Li and Dewey 2011). Finally, differentially expressed genes were identified using DEseq2 (1.4.5) (Love et al. 2014). The threshold used to classify mRNAs as robustly expressed was set at a mean normalized count of 500 across the time series to allow us to robustly detect changes in expression that may be well below the mean of the time series.

### Clustering

To cluster the dynamic responses of estrogen-regulated genes, 1D interpolation was preformed using a piecewise cubic Hermite interpolating polynomial (Fritsch and Carlson 1980) to estimate the expression at unmeasured time points. Next, the response of each gene was subjected to 1D wavelet decomposition (Mallat 1989) using a Daubechies 3 wavelet. Finally, the vectors of wavelet coefficients were hierarchically clustered and split into three clusters.

### Gene set and miRNA target site enrichment

Enrichment of the three classes of estrogen-responsive genes within GO biological processes categories was assessed using the PANTHER overrepresentation test (Mi et al. 2013). A selection of the most significant categories is depicted in Figure 2E–G. miRNA target site enrichment was conducted by (i) identifying the list of miRNA families whose members have a mean expression of 50 RPMM, (ii) identifying lists of “characteristic genes” whose change in expression best describes the difference between consecutive time points (Clark et al. 2014), and (iii) using our miRNA target site enrichment algorithm (miRhub) (Baran-Gale et al. 2013) to identify miRNA families that act as “master regulators” of the “characteristic gene sets.” miRNA target site predictions used in the miRhub enrichment algorithm are derived from TargetScan5.2 (Grimson et al. 2007).

### Real-time quantitative PCR analysis

Using total RNA from above, complementary DNA (cDNA) was synthesized using the TaqMan miRNA Reverse Transcription kit (Applied Biosystems; #4366596) according to the manufacturer's instructions, or using the High-capacity RNA-to-cDNA kit (Applied Biosystems; #4387406). Real-time PCR amplification of miRNAs was performed using TaqMan miRNA assays in TaqMan Universal PCR Master Mix (Applied Biosystems; #4304437) on a Bio-Rad CFX96 Touch Real Time PCR Detection system (Bio-Rad Laboratories, Inc.). Reactions were performed in triplicate using *RNU66* as the internal control. Real-time PCR amplification of mRNAs was performed using SsoAdvanced Universal SYBR Green Supermix (Bio-Rad Laboratories, Inc.; #1725271) on a Bio-Rad CFX96 Touch Real Time PCR Detection system (Bio-Rad Laboratories, Inc.). Reactions were performed in triplicate using *RPS9* as the internal control. All TaqMan assays used in this study were purchased from Applied Biosystems, Inc. and include *miR-503* and *RNU66*.

### Luciferase reporter analysis

We utilized a dual-luciferase reporter system (GeneCopoeia; # HmiT018728) in which the 3′-UTR of *ZNF217* was fused to the end of Firefly luciferase. The construct also contains Renilla luciferase, which can be used as an internal control. Next, we mutated the vector to interfere with the binding of the miR-503 seed region (nucleotides 2–8) using a QuikChange II XL Site-Directed Mutagenesis Kit (Agilent; #200521). Two A's were added in the miR-503 target site of ZNF217 to induce a bulge in the target site opposite of nucleotides 3 and 4 of miR-503's seed. MCF-7 cells were transiently transfected with the wild-type or mutant reporter with or cotransfected with miRIDIAN microRNA Human hsa-miR-503-5p mimic (Dharmacon #C-300841-05-0005) and with the vector. Luminescence was measured 48 h after transfection using the Luc-Pair Duo-Luciferase Assay (GeneCopoeia; # LPFR-P030) on a Promega GloMax Multi+ Detection System luminometer.

### Cell proliferation

Cell proliferation was measured using the Click-iT EdU Alexa Fluor 488 Imaging Kit (Invitrogen; # C10337). MCF-7 cells were transfected with 50 nM miR-503 mimic or with transfection reagent only and were cultured for 46 or 70 h. Cells were then pulsed with EdU for 2 h before cells were fixed according to the Click-iT protocol. Finally, EdU was labeled with Alexa Fluor 488 dye, DNA was stained with Hoechst and were imaged at 20× on an inverted fluorescence microscope with a Nikon TI Eclipse camera. Image segmentation analysis was performed using the Nikon Elements software package.

### Western blots

MCF7 cells were harvested 48 h after transfection with 50 nM miR-503 mimic or transfection reagent only (mock). Cells were lysed in RIPA buffer with protease inhibitor. Protein concentration was determined using the Pierce BCA Protein assay kit (Thermo Fisher; # 23225). Equal amounts of protein for each sample were loaded on a Mini-PROTEAN TGX gel (Bio-Rad Laboratories, Inc.). Following gel electrophoresis, proteins were transferred to nitrocellulose membrane and blocked in 5% milk at 4°C overnight. Ki67 levels were measured using the Anti-Ki67 antibody (Abcam; # ab15580). ZNF217 levels were measured using the Anti-ZNF217 antibody (Abcam; # ab48133). β-Actin levels were measured using an anti-β-actin antibody (HRP) (Abcam; # ab20272).

### Survival analysis

Overall survival of ER+ breast cancer patients based on *ZNF217* high/low status was evaluated using the Kaplan–Meier plotter (http://kmplot.com/; Győrffy et al. 2009; Frietze et al. 2014). The following parameters were used on the breast cancer database: Affy ID = 203739_at; survival = OS; split patients by = median; ER status = ER positive; derive ER status from gene expression data = checked; database version = 2014 (*n* = 4142). Overall survival of ER+ tamoxifen-treated breast cancer patients based on miR-503 high/low status was evaluated using the MiRUMIR tool (Antonov 2011; Antonov et al. 2013), using a data set from Lyng et al. (2012) (GSE37405).

## DATA DEPOSITION

RNA-seq and small RNA-seq libraries are available at the Gene Expression Omnibus (GEO) under the accession number GSE78169.

## SUPPLEMENTAL MATERIAL

Supplemental material is available for this article.

## Supplementary Material

Supplemental Material

## References

[BARAN-GALERNA056895C1] Alon U. 2007 Network motifs: theory and experimental approaches. Nat Rev Genet 8: 450–461. 17510665 10.1038/nrg2102

[BARAN-GALERNA056895C2] Antonov AV. 2011 BioProfiling.de: analytical web portal for high-throughput cell biology. Nucleic Acids Res 39: W323–W327. 21609949 10.1093/nar/gkr372 PMC3125774

[BARAN-GALERNA056895C3] Antonov AV, Knight RA, Melino G, Barlev NA, Tsvetkov PO. 2013 MIRUMIR: an online tool to test microRNAs as biomarkers to predict survival in cancer using multiple clinical data sets. Cell Death Differ 20: 367. 23175189 10.1038/cdd.2012.137 PMC3554342

[BARAN-GALERNA056895C4] Baran-Gale J, Fannin EE, Kurtz CL, Sethupathy P. 2013 β-Cell 5′-shifted isomiRs are candidate regulatory hubs in type 2 diabetes. PLoS One 8: e73240. 24039891 10.1371/journal.pone.0073240 PMC3767796

[BARAN-GALERNA056895C5] Bhat-Nakshatri P, Wang G, Collins NR, Thomson MJ, Geistlinger TR, Carroll JS, Brown M, Hammond S, Srour EF, Liu Y, 2009 Estradiol-regulated microRNAs control estradiol response in breast cancer cells. Nucleic Acids Res 37: 4850–4861. 19528081 10.1093/nar/gkp500 PMC2724297

[BARAN-GALERNA056895C6] Bourdeau V, Deschênes J, Laperrière D, Aid M, White JH, Mader S. 2008 Mechanisms of primary and secondary estrogen target gene regulation in breast cancer cells. Nucleic Acids Res 36: 76–93. 17986456 10.1093/nar/gkm945 PMC2248750

[BARAN-GALERNA056895C7] Casneuf T, Van de Peer Y, Huber W. 2007 In situ analysis of cross-hybridisation on microarrays and the inference of expression correlation. BMC Bioinformatics 8: 461. 18039370 10.1186/1471-2105-8-461 PMC2213692

[BARAN-GALERNA056895C8] Castellano L, Giamas G, Jacob J, Coombes RC, Lucchesi W, Thiruchelvam P, Barton G, Jiao LR, Wait R, Waxman J, 2009 The estrogen receptor-α-induced microRNA signature regulates itself and its transcriptional response. Proc Natl Acad Sci 106: 15732–15737. 19706389 10.1073/pnas.0906947106 PMC2747188

[BARAN-GALERNA056895C9] Chang EC, Charn TH, Park S-H, Helferich WG, Komm B, Katzenellenbogen JA, Katzenellenbogen BS. 2008 Estrogen receptors α and β as determinants of gene expression: influence of ligand, dose, and chromatin binding. Mol Endocrinol 22: 1032–1043. 18258689 10.1210/me.2007-0356 PMC2366177

[BARAN-GALERNA056895C10] Chen EY, Tan CM, Kou Y, Duan Q, Wang Z, Meirelles GV, Clark NR, Ma'ayan A. 2013 Enrichr: interactive and collaborative HTML5 gene list enrichment analysis tool. BMC Bioinformatics 14: 128. 23586463 10.1186/1471-2105-14-128 PMC3637064

[BARAN-GALERNA056895C11] Chong Y, Zhang J, Guo X, Li G, Zhang S, Li C, Jiao Z, Shao M. 2014 MicroRNA-503 acts as a tumor suppressor in osteosarcoma by targeting L1CAM. PLoS One 9: e114585. 25536034 10.1371/journal.pone.0114585 PMC4275157

[BARAN-GALERNA056895C12] Cicatiello L, Mutarelli M, Grober OMV, Paris O, Ferraro L, Ravo M, Tarallo R, Luo S, Schroth GP, Seifert M, 2010 Estrogen receptor α controls a gene network in luminal-like breast cancer cells comprising multiple transcription factors and microRNAs. Am J Pathol 176: 2113–2130. 20348243 10.2353/ajpath.2010.090837 PMC2861078

[BARAN-GALERNA056895C13] Clark NR, Hu KS, Feldmann AS, Kou Y, Chen EY, Duan Q, Ma'ayan A. 2014 The characteristic direction: a geometrical approach to identify differentially expressed genes. BMC Bioinformatics 15: 79. 24650281 10.1186/1471-2105-15-79 PMC4000056

[BARAN-GALERNA056895C14] Cohen PA, Donini CF, Nguyen NT, Lincet H, Vendrell JA. 2015 The dark side of ZNF217, a key regulator of tumorigenesis with powerful biomarker value. Oncotarget 6: 41566–41581. 26431164 10.18632/oncotarget.5893 PMC4747174

[BARAN-GALERNA056895C15] Creighton CJ, Cordero KE, Larios JM, Miller RS, Johnson MD, Chinnaiyan AM, Lippman ME, Rae JM. 2006 Genes regulated by estrogen in breast tumor cells in vitro are similarly regulated in vivo in tumor xenografts and human breast tumors. Genome Biol 7: R28. 16606439 10.1186/gb-2006-7-4-r28 PMC1557996

[BARAN-GALERNA056895C16] Eeckhoute J, Keeton EK, Lupien M, Krum SA, Carroll JS, Brown M. 2007 Positive cross-regulatory loop ties GATA-3 to estrogen receptor α expression in breast cancer. Cancer Res 67: 6477–6483. 17616709 10.1158/0008-5472.CAN-07-0746

[BARAN-GALERNA056895C17] Frankel LB, Christoffersen NR, Jacobsen A, Lindow M, Krogh A, Lund AH. 2008 Programmed cell death 4 (PDCD4) is an important functional target of the microRNA miR-21 in breast cancer cells. J Biol Chem 283: 1026–1033. 17991735 10.1074/jbc.M707224200

[BARAN-GALERNA056895C18] Frasor J, Danes JM, Komm B, Chang KCN, Lyttle CR, Katzenellenbogen BS. 2003 Profiling of estrogen up- and down-regulated gene expression in human breast cancer cells: insights into gene networks and pathways underlying estrogenic control of proliferation and cell phenotype. Endocrinology 144: 4562–4574. 12959972 10.1210/en.2003-0567

[BARAN-GALERNA056895C19] Frietze S, O'Geen H, Littlepage LE, Simion C, Sweeney CA, Farnham PJ, Krig SR. 2014 Global analysis of ZNF217 chromatin occupancy in the breast cancer cell genome reveals an association with ERα. BMC Genomics 15: 520. 24962896 10.1186/1471-2164-15-520 PMC4082627

[BARAN-GALERNA056895C20] Fritsch FN, Carlson RE. 1980 Monotone piecewise cubic interpolation. SIAM J Numer Anal 17: 238–246.

[BARAN-GALERNA056895C21] Gaube F, Wolfl S, Pusch L, Kroll TC, Hamburger M. 2007 Gene expression profiling reveals effects of *Cimicifuga racemosa* (L.) NUTT. (black cohosh) on the estrogen receptor positive human breast cancer cell line MCF-7. BMC Pharmacol 7: 11. 17880733 10.1186/1471-2210-7-11 PMC2194763

[BARAN-GALERNA056895C22] Grimson A, Farh KK-H, Johnston WK, Garrett-Engele P, Lim LP, Bartel DP. 2007 MicroRNA targeting specificity in mammals: determinants beyond seed pairing. Mol Cell 27: 91–105. 17612493 10.1016/j.molcel.2007.06.017 PMC3800283

[BARAN-GALERNA056895C23] Guo J, Liu X, Wang M. 2015 miR-503 suppresses tumor cell proliferation and metastasis by directly targeting RNF31 in prostate cancer. Biochem Biophys Res Commun 464: 1302–1308. 26231797 10.1016/j.bbrc.2015.07.127

[BARAN-GALERNA056895C24] Győrffy B, Lanczky A, Eklund AC, Denkert C, Budczies J, Li Q, Szallasi Z. 2009 An online survival analysis tool to rapidly assess the effect of 22,277 genes on breast cancer prognosis using microarray data of 1,809 patients. Breast Cancer Res Treat 123: 725–731. 20020197 10.1007/s10549-009-0674-9

[BARAN-GALERNA056895C25] Higa GM, Fell RG. 2013 Sex hormone receptor repertoire in breast cancer. Int J Breast Cancer 2013: 284036. 24324894 10.1155/2013/284036 PMC3845405

[BARAN-GALERNA056895C26] Jagannathan V, Robinson-Rechavi M. 2011 Meta-analysis of estrogen response in MCF-7 distinguishes early target genes involved in signaling and cell proliferation from later target genes involved in cell cycle and DNA repair. BMC Syst Biol 5: 138. 21878096 10.1186/1752-0509-5-138 PMC3225231

[BARAN-GALERNA056895C27] Jensen TW, Ray T, Wang J, Li X, Naritoku WY, Han B, Bellafiore F, Bagaria SP, Qu A, Cui X, 2015 Diagnosis of basal-like breast cancer using a FOXC1-based assay. J Natl Cancer Inst 107 10.1093/jnci/djv148 PMC4554196 26041837

[BARAN-GALERNA056895C28] Jiang X, Chen Y, Du E, Yang K, Zhang Z, Qi S, Xu Y. 2016 GATA3-driven expression of miR-503 inhibits prostate cancer progression by repressing ZNF217 expression. Cell Signal 28: 1216–1224. 27267060 10.1016/j.cellsig.2016.06.002

[BARAN-GALERNA056895C29] Kong SL, Li G, Loh SL, Sung W-K, Liu ET. 2011 Cellular reprogramming by the conjoint action of ERα, FOXA1, and GATA3 to a ligand-inducible growth state. Mol Syst Biol 7: 526. 21878914 10.1038/msb.2011.59 PMC3202798

[BARAN-GALERNA056895C30] Li B, Dewey CN. 2011 RSEM: accurate transcript quantification from RNA-Seq data with or without a reference genome. BMC Bioinformatics 12: 323. 21816040 10.1186/1471-2105-12-323 PMC3163565

[BARAN-GALERNA056895C31] Li J, Song L, Qiu Y, Yin A, Zhong M. 2014 ZNF217 is associated with poor prognosis and enhances proliferation and metastasis in ovarian cancer. Int J Clin Exp Pathol 7: 3038–3047. 25031722 PMC4097264

[BARAN-GALERNA056895C32] Li B, Liu L, Li X, Wu L. 2015 miR-503 suppresses metastasis of hepatocellular carcinoma cell by targeting PRMT1. Biochem Biophys Res Commun 464: 982–987. 26163260 10.1016/j.bbrc.2015.06.169

[BARAN-GALERNA056895C33] Long J, Ou C, Xia H, Zhu Y, Liu D. 2015 MiR-503 inhibited cell proliferation of human breast cancer cells by suppressing CCND1 expression. Tumour Biol 36: 8697–8702. 26047605 10.1007/s13277-015-3623-8

[BARAN-GALERNA056895C34] Love MI, Huber W, Anders S. 2014 Moderated estimation of fold change and dispersion for RNA-seq data with DESeq2. Genome Biol 15: 550. 25516281 10.1186/s13059-014-0550-8 PMC4302049

[BARAN-GALERNA056895C35] Lyng MB, Lænkholm A-V, Søkilde R, Gravgaard KH, Litman T, Ditzel HJ. 2012 Global microRNA expression profiling of high-risk ER+ breast cancers from patients receiving adjuvant tamoxifen mono-therapy: a DBCG study. PLoS One 7: e36170. 22623953 10.1371/journal.pone.0036170 PMC3356496

[BARAN-GALERNA056895C36] Mallat SG. 1989 A theory for multiresolution signal decomposition: the wavelet representation. IEEE Trans Pattern Anal Mach Intell 11: 674–693.

[BARAN-GALERNA056895C37] Martin M. 2011 Cutadapt removes adapter sequences from high-throughput sequencing reads. EMBnet J 17: 10–12.

[BARAN-GALERNA056895C38] Mi H, Muruganujan A, Casagrande JT, Thomas PD. 2013 Large-scale gene function analysis with the PANTHER classification system. Nat Protoc 8: 1551–1566. 23868073 10.1038/nprot.2013.092 PMC6519453

[BARAN-GALERNA056895C39] Mukherji S, Ebert MS, Zheng GXY, Tsang JS, Sharp PA, van Oudenaarden A. 2011 MicroRNAs can generate thresholds in target gene expression. Nat Genet 43: 854–859. 21857679 10.1038/ng.905 PMC3163764

[BARAN-GALERNA056895C40] Nguyen NT, Vendrell JA, Poulard C, Győrffy B, Goddard-Léon S, Bièche I, Corbo L, Le Romancer M, Bachelot T, Treilleux I, 2014 A functional interplay between ZNF217 and estrogen receptor α exists in luminal breast cancers. Mol Oncol 8: 1441–1457. 24973012 10.1016/j.molonc.2014.05.013 PMC5528595

[BARAN-GALERNA056895C41] Oh DS, Troester MA, Usary J, Hu Z, He X, Fan C, Wu J, Carey LA, Perou CM. 2006 Estrogen-regulated genes predict survival in hormone receptor-positive breast cancers. J Clin Oncol 24: 1656–1664. 16505416 10.1200/JCO.2005.03.2755

[BARAN-GALERNA056895C42] Plaza-Menacho I, Mologni L, McDonald NQ. 2014 Mechanisms of RET signaling in cancer: current and future implications for targeted therapy. Cell Signal 26: 1743–1752. 24705026 10.1016/j.cellsig.2014.03.032

[BARAN-GALERNA056895C43] Polioudakis D, Abell NS, Iyer VR. 2015 miR-503 represses human cell proliferation and directly targets the oncogene DDHD2 by non-canonical target pairing. BMC Genomics 16: 40. 25653011 10.1186/s12864-015-1279-9 PMC4326481

[BARAN-GALERNA056895C44] Qi L, Bart J, Tan LP, Platteel I, Sluis TVD, Huitema S, Harms G, Fu L, Hollema H, Berg AVD. 2009 Expression of miR-21 and its targets (PTEN, PDCD4, TM1) in flat epithelial atypia of the breast in relation to ductal carcinoma in situ and invasive carcinoma. BMC Cancer 9: 163. 19473551 10.1186/1471-2407-9-163 PMC2695476

[BARAN-GALERNA056895C45] Rae JM, Johnson MD, Scheys JO, Cordero KE, Larios JM, Lippman ME. 2005 GREB 1 is a critical regulator of hormone dependent breast cancer growth. Breast Cancer Res Treat 92: 141–149. 15986123 10.1007/s10549-005-1483-4

[BARAN-GALERNA056895C46] Rao X, Di Leva G, Li M, Fang F, Devlin C, Hartman-Frey C, Burow ME, Ivan M, Croce CM, Nephew KP. 2011 MicroRNA-221/222 confers breast cancer fulvestrant resistance by regulating multiple signaling pathways. Oncogene 30: 1082–1097. 21057537 10.1038/onc.2010.487 PMC3342929

[BARAN-GALERNA056895C47] Reid G, Hübner MR, Métivier R, Brand H, Denger S, Manu D, Beaudouin J, Ellenberg J, Gannon F. 2003 Cyclic, proteasome-mediated turnover of unliganded and liganded ERα on responsive promoters is an integral feature of estrogen signaling. Mol Cell 11: 695–707. 12667452 10.1016/s1097-2765(03)00090-x

[BARAN-GALERNA056895C48] Sabatier R, Gonçalves A, Bertucci F. 2014 Personalized medicine: present and future of breast cancer management. Crit Rev Oncol Hematol 91: 223–233. 24725667 10.1016/j.critrevonc.2014.03.002

[BARAN-GALERNA056895C49] Shang Y, Hu X, DiRenzo J, Lazar MA, Brown M. 2000 Cofactor dynamics and sufficiency in estrogen receptor-regulated transcription. Cell 103: 843–852. 11136970 10.1016/s0092-8674(00)00188-4

[BARAN-GALERNA056895C50] Simonini P, Breiling A, Gupta N, Malekpour M. 2010 Epigenetically deregulated microRNA-375 is involved in a positive feedback loop with estrogen receptor α in breast cancer cells. Cancer Res 70: 9175–9184. 20978187 10.1158/0008-5472.CAN-10-1318

[BARAN-GALERNA056895C51] Smith CL, Migliaccio I, Chaubal V, Wu M-F, Pace MC, Hartmaier R, Jiang S, Edwards DP, Gutiérrez MC, Hilsenbeck SG, 2012 Elevated nuclear expression of the SMRT corepressor in breast cancer is associated with earlier tumor recurrence. Breast Cancer Res Treat 136: 253–265. 23015261 10.1007/s10549-012-2262-7 PMC3511772

[BARAN-GALERNA056895C52] Sørlie T, Perou CM, Tibshirani R, Aas T, Geisler S, Johnsen H, Hastie T, Eisen MB, van de Rijn M, Jeffrey SS, 2001 Gene expression patterns of breast carcinomas distinguish tumor subclasses with clinical implications. Proc Natl Acad Sci 98: 10869–10874. 11553815 10.1073/pnas.191367098 PMC58566

[BARAN-GALERNA056895C53] Suter R, Marcum JA. 2007 The molecular genetics of breast cancer and targeted therapy. Biologics 1: 241–258. 19707334 PMC2721311

[BARAN-GALERNA056895C54] Thollet A, Vendrell JA, Payen L, Ghayad SE, Ben Larbi S, Grisard E, Collins C, Villedieu M, Cohen PA. 2010 ZNF217 confers resistance to the pro-apoptotic signals of paclitaxel and aberrant expression of Aurora-A in breast cancer cells. Mol Cancer 9: 291. 21059223 10.1186/1476-4598-9-291 PMC2996367

[BARAN-GALERNA056895C55] Tkocz D, Crawford NT, Buckley NE, Berry FB, Kennedy RD, Gorski JJ, Harkin DP, Mullan PB. 2011 BRCA1 and GATA3 corepress FOXC1 to inhibit the pathogenesis of basal-like breast cancers. Oncogene 31: 3667–3678. 22120723 10.1038/onc.2011.531

[BARAN-GALERNA056895C56] Usary J, Llaca V, Karaca G, Presswala S, Karaca M, He X, Langerød A, Kåresen R, Oh DS, Dressler LG, 2004 Mutation of GATA3 in human breast tumors. Oncogene 23: 7669–7678. 15361840 10.1038/sj.onc.1207966

[BARAN-GALERNA056895C57] Wang K, Singh D, Zeng Z, Coleman SJ, Huang Y, Savich GL, He X, Mieczkowski P, Grimm SA, Perou CM, 2010 MapSplice: accurate mapping of RNA-seq reads for splice junction discovery. Nucleic Acids Res 38: e178. 20802226 10.1093/nar/gkq622 PMC2952873

[BARAN-GALERNA056895C58] Wiese TE, Kral LG, Dennis KE, Butler WB, Brooks SC. 1992 Optimization of estrogen growth response in MCF-7 cells. In Vitro Cell Dev Biol 28A: 595–602. 1429362 10.1007/BF02631033

[BARAN-GALERNA056895C59] Yin Z-L, Wang Y-L, Ge S-F, Guo T-T, Wang L, Zheng X-M, Liu J. 2015 Reduced expression of miR-503 is associated with poor prognosis in cervical cancer. Eur Rev Med Pharmacol Sci 19: 4081–4085. 26592830

[BARAN-GALERNA056895C60] Zhou W, Slingerland JM. 2014 Links between oestrogen receptor activation and proteolysis: relevance to hormone-regulated cancer therapy. Nat Rev Cancer 14: 26–38. 24505618 10.1038/nrc3622

[BARAN-GALERNA056895C61] Zhu S, Si M-L, Wu H, Mo Y-Y. 2007 MicroRNA-21 targets the tumor suppressor gene tropomyosin 1 (TPM1). J Biol Chem 282: 14328–14336. 17363372 10.1074/jbc.M611393200

[BARAN-GALERNA056895C62] Zhu S, Wu H, Wu F, Nie D, Sheng S, Mo Y-Y. 2008 MicroRNA-21 targets tumor suppressor genes in invasion and metastasis. Cell Res 18: 350–359. 18270520 10.1038/cr.2008.24

