# Supplementary material for: An integrative transcriptomics approach identifies miR-503 as a candidate master regulator of the estrogen response in MCF-7 breast cancer cells
Source: RNA. 2016 Oct;22(10):1592–603. doi: 10.1261/rna.056895.116 (PMC5029456; doi:10.1261/rna.056895.116)
Supplement: Supplemental Material [file supp_22_10_1592__index.html]

An integrative transcriptomics approach identifies miR-503 as a candidate master regulator of the estrogen response in MCF-7 breast cancer cells — Supplemental Material 

# An integrative transcriptomics approach identifies miR-503 as a candidate master regulator of the estrogen response in MCF-7 breast cancer cells

## Supplemental Material

- Supplemental\_FigS1-S7.pdf
- Supplemental\_Legends.docx
- Supplemental\_TableS1-S4.xlsx
