# Supplementary material for: An integrative transcriptomics approach identifies miR-503 as a candidate master regulator of the estrogen response in MCF-7 breast cancer cells
Source: RNA. 2016 Oct;22(10):1592–603. doi: 10.1261/rna.056895.116 (PMC5029456; doi:10.1261/rna.056895.116)
Supplement: Supplemental Material [file supp_056895.116_Supplemental_FigS1-S7.pdf]

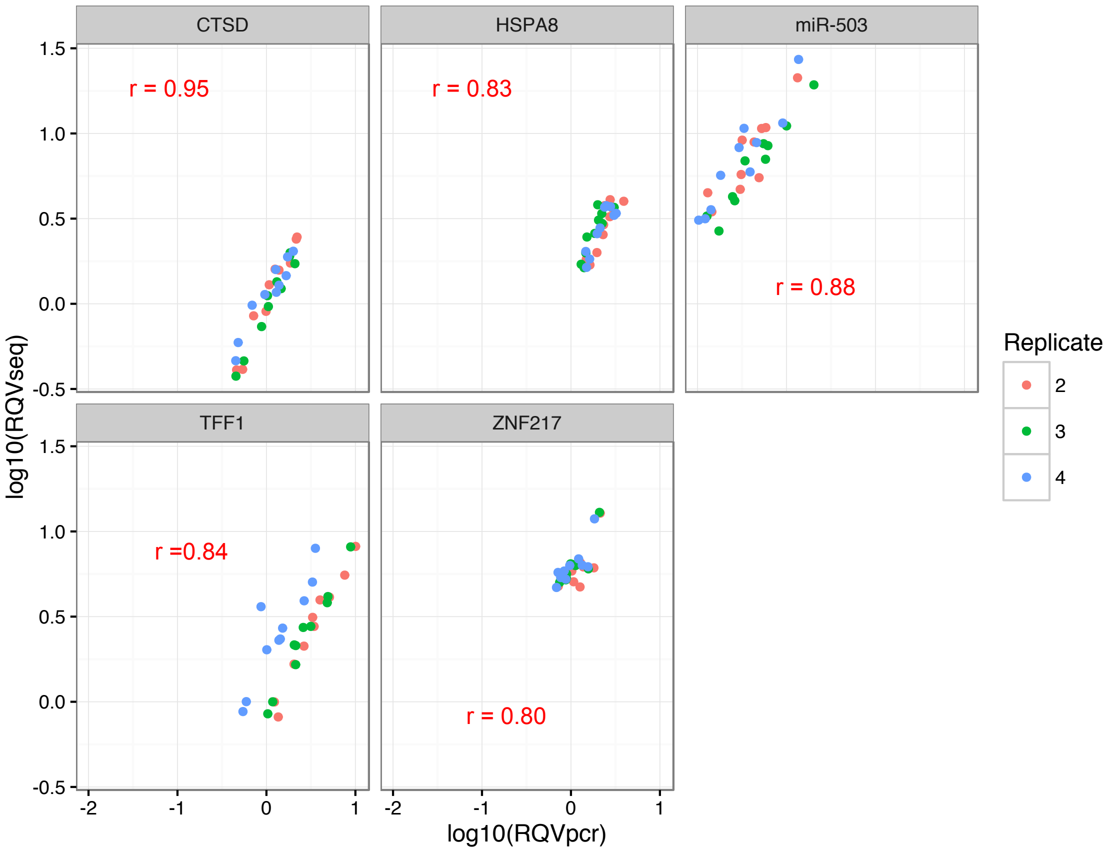

Figure S1: PCR validation of selected RNAs. Comparison between the relative quantitative value (RQV) of selected RNAs as measured by high throughput sequencing (y-axis) or RT-qPCR (x-axis). Pearson's correlation value (r) is shown in red on each plot.

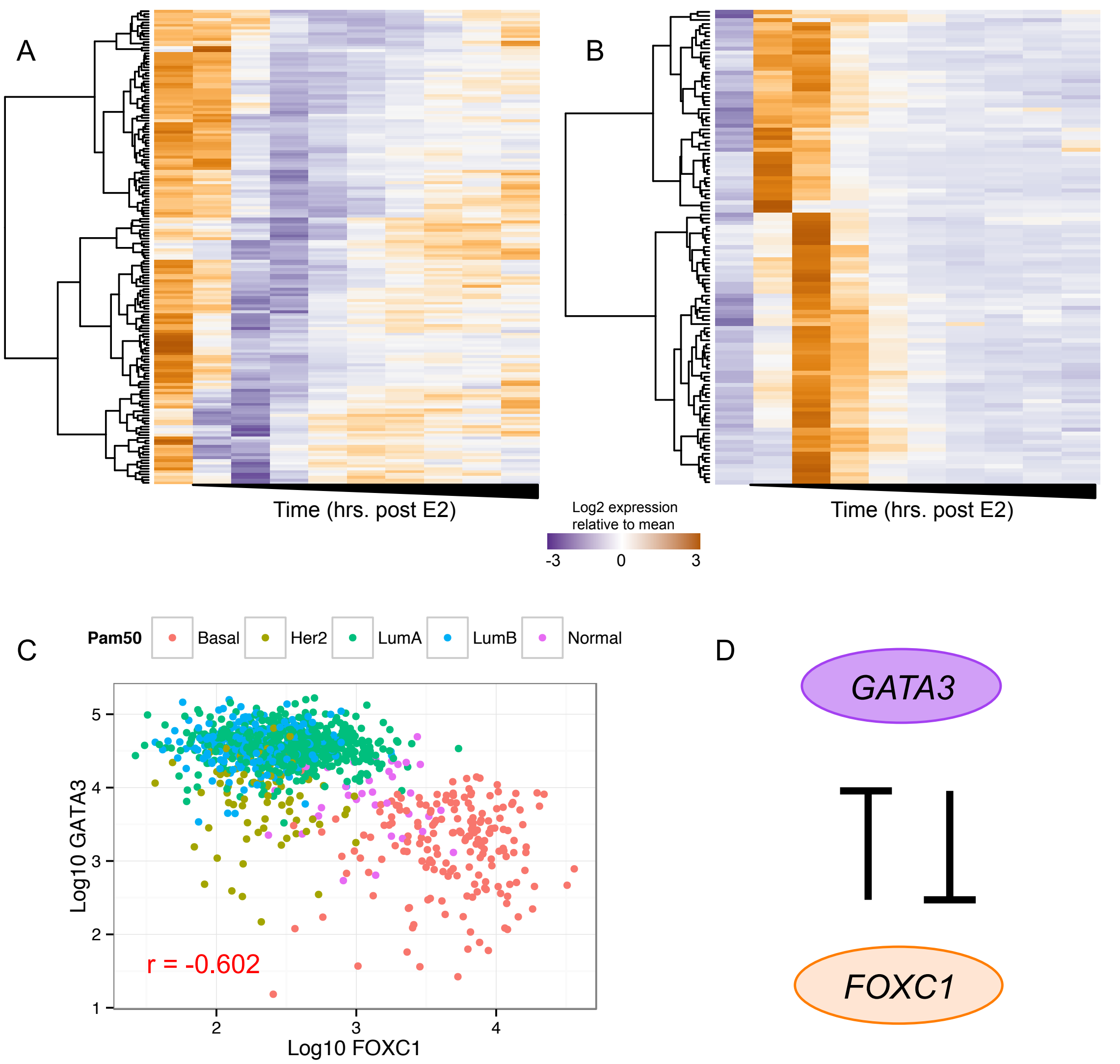

Figure S2: *GATA3*-like and anti-*GATA3*-like genes. (A) The expression profile of 170 mRNAs that exhibit an expression pattern similar to *GATA3*. (B) The expression profile of 118 mRNAs that exhibit a pattern of expression opposite of that of *GATA3*. (C) Normalized expression of *GATA3* and *FOXC1* in 1046 samples from The Cancer Genome Atlas breast cancer dataset. Pearson's correlation (r) is shown in red. (D) Opposite expression pattern of *GATA3* and *FOXC1* is consistent with a mutual inhibition relationship.

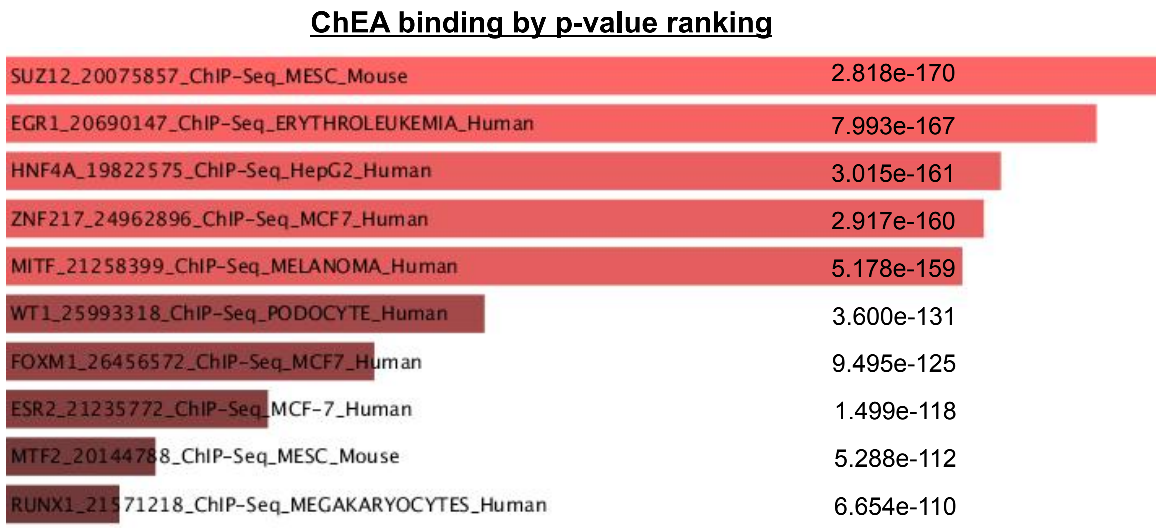

Figure S3: Top ChEA binding hits for estrogen responsive mRNAs. The ten most enriched transcription factor binding sites for estrogen responsive mRNAs in our dataset using ChEA. Transcription factors are sorted by p-value ranking.

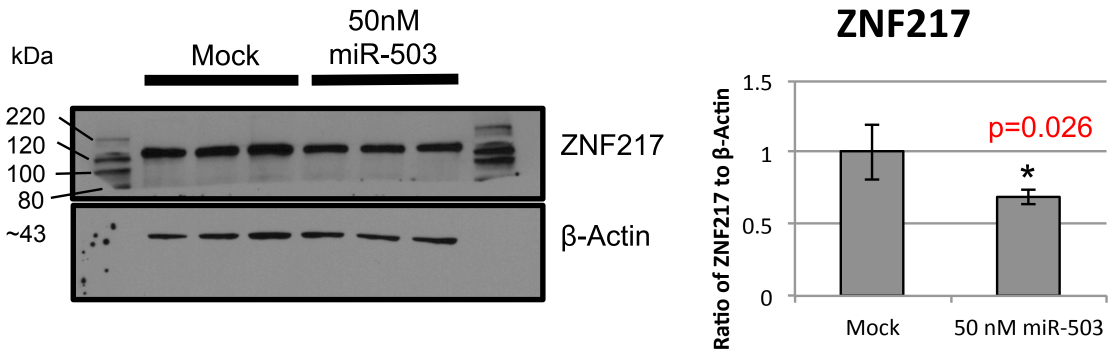

Figure S4: ZNF217 protein levels in Mock and miR-503 transfected MCF-7 cells. Western blot showing ZNF217 protein levels in Mock or miR-503 (50nM) transfected MCF-7 cells at 48 hours post transfection. Significance assessed using a 1-tailed Student's t-test.

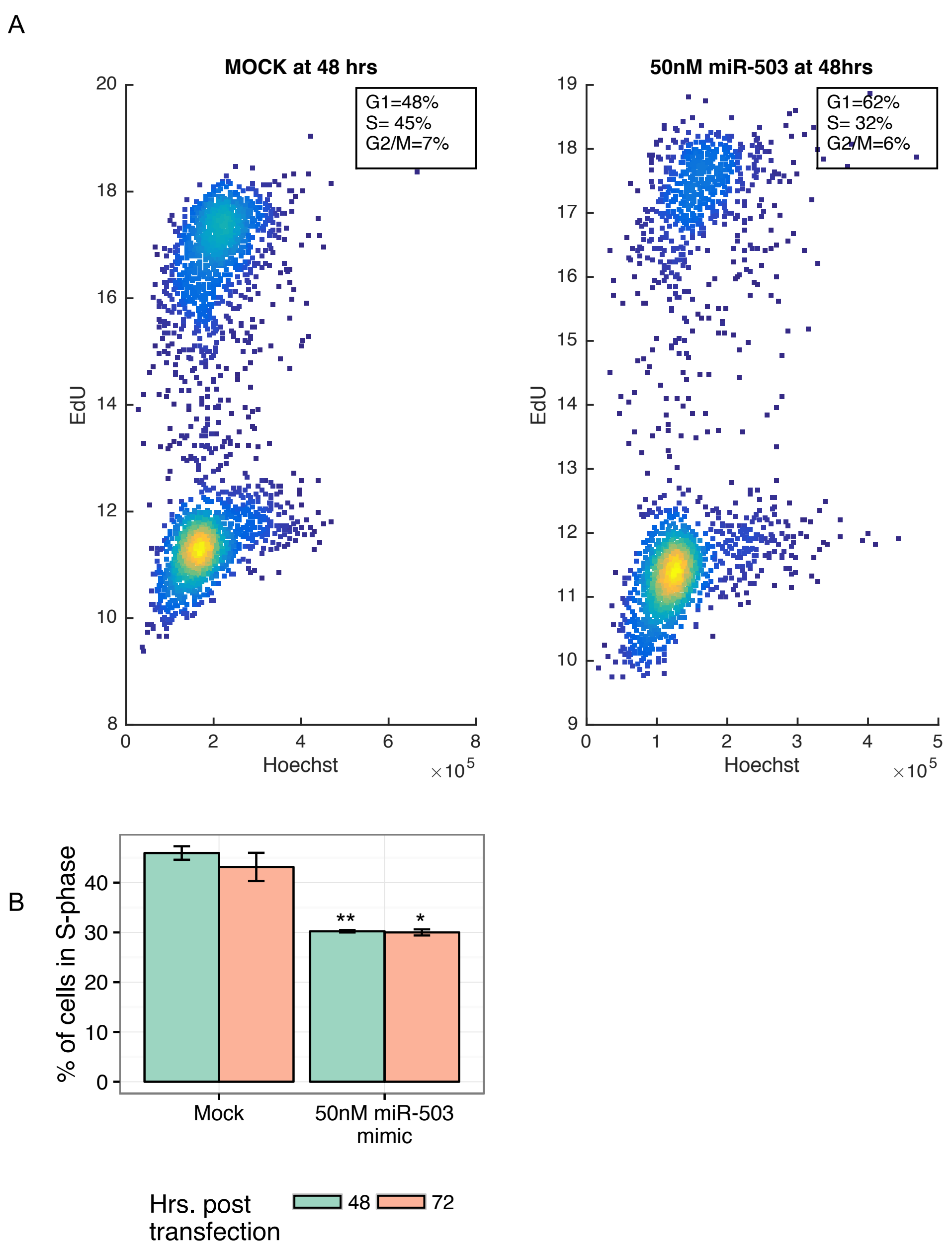

Figure S5: miR-503/ZNF217 circuit inhibits proliferation. (A) Total EdU and DNA (Hoechst) fluorescence in cells from Mock or miR-503 (50nM) transfected MCF7 cells. (B) The percentage of MCF7 cells in S-phase during the EdU pulse experiment form Mock or miR-503 (50nM) transfected MCF7 cells at 48 or 72 hours post transfection. Significance assessed using a Student's t-test (* P< 0.05, **P< 0.01).

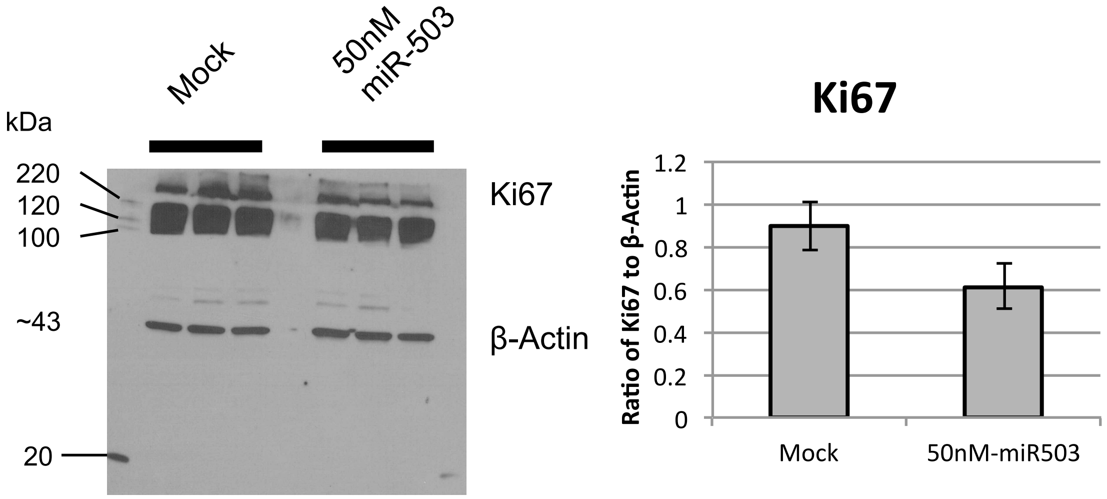

Figure S6: Ki67 protein levels in Mock and miR-503 transfected MCF7 cells. Western blot showing Ki67 protein levels in Mock or miR-503 (50nM) transfected MCF7 cells at 72 hours post transfection.

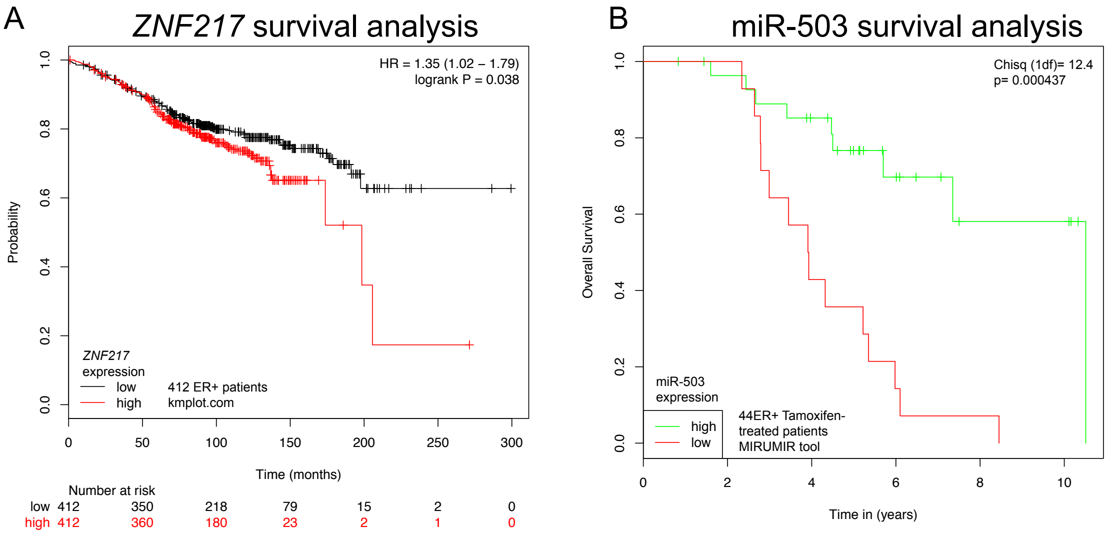

Figure S7: Association of *ZNF217* and miR-503 expression in breast cancer patient cohorts. (A) Survival analysis of 412 ER+ patients based on *ZNF217* high/low status (Frietze et al. 2014; Győrffy et al. 2009). (B) Survival analysis of 44 ER+ tamoxifen treated patients based on miR-503 high/low status (Antonov et al. 2013; Lyng et al. 2012; Antonov 2011).
