# Supplementary material for: An integrative transcriptomics approach identifies miR-503 as a candidate master regulator of the estrogen response in MCF-7 breast cancer cells
Source: RNA. 2016 Oct;22(10):1592–603. doi: 10.1261/rna.056895.116 (PMC5029456; doi:10.1261/rna.056895.116)
Supplement: Supplemental Material [file supp_056895.116_Supplemental_Legends.docx]

**Figure S1:** **PCR validation of selected RNAs.** Comparison between the relative quantitative value (RQV) of selected RNAs as measured by high throughput sequencing (y-axis) or RT-qPCR (x-axis). Pearson’s correlation value (r) is shown in red on each plot.

**Figure S2:** ***GATA3*-like and anti-*GATA3*-like genes.** (A) The expression profile of 170 mRNAs that exhibit an expression pattern similar to *GATA3*. (B) The expression profile of 118 mRNAs that exhibit a pattern of expression opposite of that of *GATA3*. (C) Normalized expression of *GATA3* and *FOXC1* in 1046 samples from The Cancer Genome Atlas breast cancer dataset. Pearson’s correlation (r) is shown in red. (D) Opposite expression pattern of *GATA3* and *FOXC1* is consistent with a mutual inhibition relationship.

**Figure S3:** **Top** **ChEA binding hits for estrogen responsive mRNAs.** The ten most enriched transcription factor binding sites for estrogen responsive mRNAs in our dataset using ChEA. Transcription factors are sorted by p-value ranking.

**Figure S4:** **ZNF217 protein levels in Mock and miR-503 transfected MCF-7 cells.** Western blot showing ZNF217 protein levels in Mock or miR-503 (50nM) transfected MCF-7 cells at 48 hours post transfection. Significance assessed using a 1-tailed Student’s t-test.

**Figure S5:** **miR-503/ZNF217 circuit inhibits proliferation.** (A) Total EdU and DNA (Hoechst) fluorescence in cells from Mock or miR-503 (50nM) transfected MCF-7 cells. (B) The percentage of MCF-7 cells in S-phase during the EdU pulse experiment form Mock or miR-503 (50nM) transfected MCF-7 cells at 48 or 72 hours post transfection. Significance assessed using a Student’s t-test (* P< 0.05, **P< 0.01).

**Figure S6:** **Ki67 protein levels in Mock and miR-503 transfected MCF-7 cells.** Western blot showing Ki67 protein levels in Mock or miR-503 (50nM) transfected MCF-7 cells at 72 hours post transfection.

**Figure S7:** **Association of *ZNF217* and miR-503 expression in breast cancer patient cohorts.** (A) Survival analysis of 412 ER+ patients based on *ZNF217* high/low status (Frietze et al. 2014; Győrffy et al. 2009). (B) Survival analysis of 44 ER+ tamoxifen treated patients based on miR-503 high/low status (Antonov et al. 2013; Lyng et al. 2012; Antonov 2011).

**Table S1: Statistics for high throughput sequencing.** This table displays the mapping statistics for both RNA-seq and small RNA-seq libraries for the 30 samples sequenced. RNA-seq statistics are collected from the MapSplice algorithm, and small RNA-seq statistics include successfully trimmed reads (3’-adapter removed), reads too short to align (trimmed length < 14), reads that perfectly align to the genome, reads that fail to align perfectly but may align with mismatches allowed, the total # of reads to align (perfectly or imperfectly), the read that align to annotated miRNA loci, and finally the read that align to tRNA loci.

**Table S2: Estrogen responsive mRNAs.** The expression profile of 1546 mRNAs that have a significant (adjusted p-value <0.05) ≥2-fold changes at one or more time points in response to estrogen stimulation are listed in this table. For each gene, the assigned cluster and the normalized expression at each time point is reported. Genes clustered into three classes (Transient, Repressed, and Induced).

**Table S3: Estrogen responsive miRNAs.** This table reports the normalized expression (RPMM) of 308 mRNAs that are expressed during the estrogen response in MCF-7 cells. miRNAs that have a significant (p-value <0.05) ≥1.5-fold changes at one or more time points in response to estrogen stimulation are highlighted in green.

**Table S4: Characteristic Directions analysis.** This table reports the direction cosine coefficient for the normal vector to the hyper plane that separates gene expression in each time interval listed. Genes are sorted by maximum absolute value of the coefficient across all time intervals.
